# Common and rare exonic *MUC5B* variants associated with type 2 diabetes in Han Chinese

**DOI:** 10.1371/journal.pone.0173784

**Published:** 2017-03-27

**Authors:** Guanjie Chen, Zhenjian Zhang, Sally N. Adebamowo, Guozheng Liu, Adebowale Adeyemo, Yanxun Zhou, Ayo P. Doumatey, Chuntao Wang, Jie Zhou, Wenqiang Yan, Daniel Shriner, Fasil Tekola-Ayele, Amy R. Bentley, Congqing Jiang, Charles N. Rotimi

**Affiliations:** 1 Center for Research on Genomics and Global Health, National Human Genome Research Institute, National Institutes of Health, Bethesda, Maryland, United States of America; 2 Suizhou Central Hospital, Suizhou, Hubei, China; Kunming Institute of Zoology, Chinese Academy of Sciences, CHINA

## Abstract

Genome-wide association studies have identified over one hundred common genetic risk variants associated with type 2 diabetes (T2D). However, most of the heritability of T2D has not been accounted for. In this study, we investigated the contribution of rare and common variants to T2D susceptibility by analyzing exome array data in 1,908 Han Chinese genotyped with Affymetrix Axiom® Exome Genotyping Arrays. Based on the joint common and rare variants analysis of 57,704 autosomal SNPs within 12,244 genes using Sequence Kernel Association Tests (SKAT), we identified significant associations between T2D and 25 variants (9 rare and 16 common) in *MUC5B*, *p*-value 1.01×10^−14^. This finding was replicated (p = 0.0463) in an independent sample that included 10,401 unrelated individuals. Sixty-six of 1,553 possible haplotypes based on 25 SNPs within *MUC5B* showed significant association with T2D (Bonferroni corrected p values < 3.2×10^−5^). The expression level of *MUC5B* is significantly higher in pancreatic tissues of persons with T2D compared to those without T2D (p-value = 5×10^−5^). Our findings suggest that dysregulated MUC5B expression may be involved in the pathogenesis of T2D. As a strong candidate gene for T2D, *MUC5B* may play an important role in the mechanisms underlying T2D etiology and its complications.

## Introduction

Type 2 Diabetes (T2D) is a growing global health problem. Currently, about 415 million people worldwide have diabetes. By 2040, the number of people living with diabetes is expected to increase to 642 million, with two-thirds of all cases occurring in low to middle-income countries[1]. In China, the prevalence of T2D increased exponentially over the past three decades. In 1980, the prevalence of T2D in China was less than 1%; this estimate increased to about 12% in 2010[2]. By 2013, there were about 114 million people with diabetes and about 500 million people with prediabetes in China. This rapid increase, which is unlike the transition that occurred in Western countries, coincided with economic growth, urbanization, changes in lifestyle and demographic characteristics in China.

In addition to the well-recognized influence of lifestyle factors on the risk of T2D, genetic factors play a major role in susceptibility to T2D. The successful application of genome wide association studies (GWAS) has provided some insight into the genetic basis of T2D. Until recently, it was generally assumed that common diseases such as T2D were caused by common variants[3]. Given that GWAS provided genotypic information on common variants, it appeared to be the ideal technique to identify variants. To date, over 100 common genetic risk variants with small effect sizes have been identified from GWAS and shown to be associated with T2D. However, the joint effects of these variants accounts for less than 10% of the heritability for T2D[4]. In this study, we examined the association of rare variants with T2D among a population of unrelated Chinese adults. Given that susceptibility to T2D likely involves the contribution of both common and rare variants, we conducted joint analysis of common and rare variants of about 58,000 autosomal SNPs.

## Materials and methods

### Study population

The China America Diabetes Mellitus (CADM) study is a large-scale genetic epidemiology study designed to investigate the genetic and environmental determinants of metabolic disorders including T2D, dyslipidemia, kidney disease, and hypertension. In CADM, ~2000 unrelated participants with written informed consent were enrolled from Suizhou, China, of whom 1908 were genotyped and included in these analyses. Suizhou, a historic city, is located in the Hubei province, central China and has a population of over 2 million, most of whom are Han Chinese (99.2%). Ethical approval for the study was obtained from the Institutional Review Boards of Howard University, the National Institutes of Health, and IRB of Suizhou Central Hospital, Suizhou, China. All enrolled participants provided written informed consent during the clinical visit before commencement of data collection by interview and collection of biospeciments. Details of the study protocol were clearly explained to each participants and potential participants had the opportunity to ask questions before signing the consent documents.

### Phenotype definitions

During a clinic examination, interviewers collected demographic information from the participants. All enrolled individuals self-identified as Han Chinese. Weight was measured in light clothes on an electronic scale to the nearest 0.1 kg, and height was measured with a stadiometer to the nearest 0.1 cm. Body mass index (BMI) was computed as weight (kg) divided by the square of height (m^2^). Blood samples were obtained from all participants after an overnight fast. T2D diagnosis was based on any of the following criteria established by the American Diabetes Association Expert Committee: fasting plasma glucose concentration ≥ 126 mg/dl (7.0 mmol/l), 2-hour post load value in the oral glucose tolerance test ≥ 200 mg/dl (11.1 mmol/l) on more than one occasion, history of T2D or on prescribed medication for diabetes. Cases were defined as individuals diagnosed with T2D, while controls were individuals without T2D. Hypertension was defined as systolic blood pressure (SBP) ≥ 140 mmHg and/or diastolic blood pressure (DBP) ≥ 90 mmHg, or use of blood pressure medication.

### DNA sample preparation, genotyping, and quality control

DNA was extracted from buffy coat samples using a chemagenic DNA Isolation Kit (PerkinElmer Chemagen Technologie Gmb, Baesweiler, Germany) following the manufacturer's instructions. Samples were genotyped using Affymetrix Axiom® Exome Genotyping Arrays. This array is primarily designed to detect coding variation and contains over 300,000 markers, including non-synonymous and synonymous SNPs as well as variants in splice and stop codons, and 30,000 single-base and complex indels. Genotypes were called using Axiom GT1 algorithm as implemented in Affymetrix genotyping console 4.1.3, which is a new genotyping procedure developed specifically for use with Affymetrix Axiom^®^ Genome-wide human arrays.

All arrays passed plate quality control following the manufacturer’s recommendations. The genotyping concordance rate (evaluated using 16 SNPs that were blind-genotyped twice) was 99.64%. The concordance rate for 10 individuals that were typed twice on the entire array was 98.64%. Of the 290,890 markers on the array, 178,943 were monomorphic, 23,756 had genotyping call rate less than 0.95 and 1,458 markers failed HWE (p value < 10^−6^). Of the remaining 86,733 markers, 85,009 were autosomal; 27,305 of the autosomal markers were removed for having minor allele counts less than 5. In all, 57,704 autosomal markers were carried forward for analysis in this study. Of these markers, 12,329 (21.37%) had a minor allele frequency (MAF) < 0.01, and 45,375 (78.63%) had MAF ≥ 0.01 (with 32,638 with MAF ≥ 0.05). A variant was classified as “common” if $MAF>\frac{1}{\sqrt{2n}}$ and “rare” if $MAF\leq \frac{1}{\sqrt{2n}}$ (n is number of individuals) = 0.0162[5]. Based on *hg19* genome build 37 (GRCh37), the 57,704 markers were located within 12,244 gene regions.

### Statistical analysis

To minimize the potential effect of population structure, we adjusted all analyses by the first two principal components (PC1 and PC2) obtained from R package, SNPRelate [6], which generates genetic covariance matrix followed by the extraction of eigenvalues and eigenvectors for the calculation of PCs. Single marker analysis for Common SNPs was implemented in PLINK [7] under a genetic additive model, adjusting for sex, age, BMI, Hypertension, and first two PCs. A permutation procedure was used to generate significance levels empirically to deal with rare alleles and small sample size[7]. Simple label swapping of phenotype (T2D) was used for 100,000,000 permutation tests. The empirical permutation p value (Emp) was pointwise and was calculated by Emp = $\frac{E+1}{N+1}$, where *E* is number of statistic values ≥ observed statistic value, and *N* is the total number of permutation.

Gene-base analyses of rare variants only and of joint common and rare variants were conducted using Sequence Kernel Association Test (SKAT)[5], with models adjusted as in the common single marker analysis. The overall joint effect of rare and common variants by gene regions was tested by combining the test statistics directly using weighted-sum statistics, *Q*_∅,*p*1,*p*2_ = (1 − ∅)*Q*_*rare*_ + ∅ *Q*_*common*_ with $\emptyset =\frac{SD[Q_{rare}]}{SD[Q_{rare}]+SD[Q_{common}]}$, given (∅,*p*_1_,*p*_2_). As rare variants are assumed to have larger effect sizes., different weight functions were used for rare and common variants as follows: *βeta*(*MAF*,*α* = 1,*β* = 25) for rare, and *βeta*(*MAF*,*α* = 0.5,*β* = 0.5) for common variants. Under null, the distribution of *Q*_∅,*p*1,*p*2_ is a mixture of $x_{1}^{2}$ distributions. These $x_{1}^{2}$ distributions are independent and identically distributed chi-square random variables with 1-degree freedom. An asymptotic p value was then computed with Davies’ method or moment matching[5]. The genome-wide and suggestive significant threshold were established as α of 2.5 × 10^−6^ and α of 2.5 × 10^−5^ respectively[8].

### Haplotype phasing and analysis

Haplotype phasing of SNPs was performed with the BEAGLE program[9], which uses the hidden Markov model (HMM) to find the most likely haplotype pair for each individual, conditional on that individual’s genotypes. Haplotype phasing was conducted on the set of 25 SNPs T2D-associated *MUC5B* SNPs. Haplotypes were tested in a logistic regression model that included age, sex, BMI, hypertension status, and first two PCs as covariates. A total of 1,553 possible haplotypes across *MUC5B* were tested. Bonferroni correction was used to adjust for multiple tests (0.05/number of possible haplotype = 3.2×10^−5^).

### Replication analysis

Replication analysis was performed in 10,401 African ancestry samples obtained from the Atherosclerosis Risk in Communities (ARIC, n = 3,137) [10], the Cleveland Family Study (CFS, n = 653) [11], the Howard University Family Study (HUFS, n = 1,976) [12], Jackson Heart Study (JHS, n = 2,187) [13], Multi-Ethnic Study of Atherosclerosis (MESA, n = 1,611) [14], and Africa America Diabetes Mellitus Study (AADM, n = 1802) [15]. Analysis was conducted using human genomic reference (hg19) coordinates. LiftOver (https://genome.ucsc.edu/cgi-bin/hgLiftOver) was used to convert genome coordinates and genome annotation between assemblies. Rare and common variants were defined as in the discovery study. The set of 25 rare and common SNPs associated with T2D in the discovery study were extracted from replication datasets. Sixteen (13 common and 3 rare variants) of 25 SNPs were available for joint common and rare variants analysis in SKAT. As in the discovery analysis, sex, age, BMI, first two principal components (PC1, and PC2) were included as covariates.

Replication of published GWAS findings was attempted using two strategies, 1) exact and local (*i*.*e*., SNPs in Linkage disequilibrium [LD] with the reported SNP[16]) for those gene regions that contained only common variants; and 2) a gene-level approach for gene regions containing both common and rare variants using SKAT. HapMap CHB reference data for Chinese ancestry populations was used for the identification of markers in LD with published variants. To adequately account for multiple testing, we estimated the effective degrees of freedom (df) for the spectrally-decomposed covariance matrix for the block of markers using this study’s (CADM) genotype data as previously described[17].

### Microarray analysis of human islets

Data was extracted from publicly-available MIAME compliant gene expression data (GEO, accession number GSE25724; GDS3882; http://www.ncbi.nlm.nih.gov), using the R package, GEOquery. The original data was generated from the analysis of islets of Langerhans isolated from T2D and non-T2D organ donors[18]. RNA was biotinylated, fragmented, and hybridized onto Affymetrix Human Genome U133A Array chips. The expression data was scanned and log_2_ normalized, and the differential gene expression between T2D and non-T2D samples was assessed. Two-tailed tests were used, and p values lower than 0.01 were considered as differentially-expressed[18].

## Results

Characteristics of study participants are displayed in Table 1. In this case-controls study of 1,908 individuals, about 50% of the cases and controls were female. Cases were older, heavier and, as expected, had significantly higher mean fasting blood glucose levels. Also, the cases had higher mean systolic and diastolic blood pressure and higher prevalence of hypertension compared to the controls (63.7% vs 39.56%, respectively).

**Table 1:** Characteristics of the study participants by type 2 diabetes status.

	T2D	Non-T2D	*P*-values **
N *	917 (48.06%)	991 (51.94%)	0.0902
Female *	454 (49.51%)	489 (49.34%)	0.9425
Age (yrs)	56.13 (9.80)	51.65 (9.24)	< 0.0001
BMI (kg/m^2^)	24.58 (3.10)	23.95 (2.95)	< 0.0001
Systolic Blood Pressure (mmHg)	137.4 (20.32)	128.2 (18.61)	< 0.0001
Diastolic Blood Pressure (mmHg)	86.17 (11.23)	82.17 (12.29)	< 0.0001
Hypertension *	584 (63.69%)	392 (39.56%)	< 0.0001
Glucose (mg/dL)	169.2 (62.20)	89.32 (9.72)	< 0.0001
Current Smokers *	194 (21.16%)	208(20.99%)	0.9288
Duration of T2D (yrs)	4.66 (4.85)	NA	

*numbers (percentage), other numbers are means and standard deviations

hypertension was defined as SBP ≥ 140 mmHg or DBP ≥ 90 mmHg or use of blood pressure medication.

** Significant level defined as ≤ 0.05.

**Fig 1:**
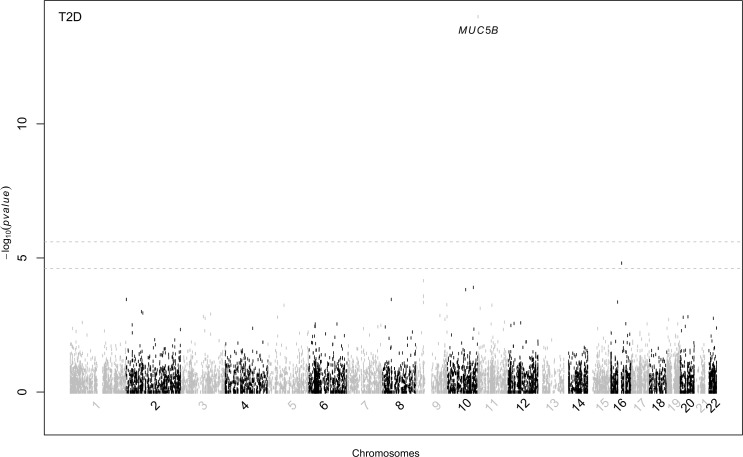
Exome Array Association Results. The y axis represents the–log_10_ (p-value) and the x axis is variant positions by chromosome. Genome-wide and suggestive statistical significance thresholds are illustrated by the two dotted lines.

In the joint common and rare variant analysis (12,244 genes), we observed a significant association between T2D and variants in the *MUC5B* gene (mucin 5B, oligomeric mucus/gel-forming, GeneID: 727897, 11p15.5) with *p*-value of 1.01 × 10^−14^ (Table 2; Fig 1 and QQ plot S1 Fig). This analysis included nine rare and sixteen common variants in *MUC5B* (Table 2). Adjustment for smoking strengthened the association (p-value = 6.29 × 10^−15^). Replication analysis was conducted in 10,401 African ancestry individuals (S1 Table) using 16 available SNPs (3 rare and 13 common) of the 25 SNPs in the *MUC5B* gene (S2 Table). The *MUC5B* finding replicated in this large sample of individuals (p = 0.0463). In CADM, the frequency of the T allele in one of the rare variants (rs12282798, MAF = 0.0047) was 0.011 among cases and < 0.001 among controls with an empirical *p*-value of 1.85 × 10^−4^ (Table 3). Four common SNPs (rs201894106 allele T, rs199967813 allele A, rs192744525 allele A, and rs199285958 allele C) with allele frequencies < 0.01 in cases, and > 0.04 in controls, statistically significant difference (empirical p-value of 10^−8^). The complete list of allele counts within *MUC5B* by T2D status and associated p-values obtained from permutation (n = 10^8^) tests are presented in Table 3.

**Table 2:** Top results for the joint association analyses of common and rare exome variants with T2D in Han Chinese individuals.

Genes	Regions	*P*-values *	# of SNPs	# of Rare	# of Common
*MUC5B*	11p15.5	1.01E-14	25	9	16
*ABCC12*	16q12.1	1.56E-5	3	2	1

* Genome-wide statistical significance threshold (p value < 2.5×10^−6^) and suggestive genome wide significant threshold (p < 2.5 ×10^−5^).

**Table 3:** Allele Counts by type 2 diabetes status for variants in the *MUC5B and ABCC12 genes*.

							Allele count				
							T2D		Non T2D		
*GENES*	SNPs	PPS	Allele 1	Allele 2	MAF	HWE	Allele 1	Allele 2	Allele 1	Allele 2	Emp **
*MUC5B*	rs2075853	1247458	T	C	0.3337	0.9418	630	1188	634	1336	0.9165
	rs80298911	1256409	A	G	0.0055 *	1.0000	12	1822	9	1959	1
	rs200226020	1261561	T	C	0.0042 *	1.0000	4	1828	12	1964	0.8113
	rs201894106	1262540	T	C	0.0284	0.1632	12	1812	96	1884	1e-08
	rs1541314	1263523	A	G	0.0625	0.6151	104	1724	134	1846	0.9905
	rs2943510	1263776	T	C	0.0661	1.0000	112	1718	140	1842	0.9833
	rs61997210	1264292	T	C	0.0045 *	1.0000	10	1814	7	1975	1
	rs12282798	1266617	T	C	0.0047 *	1.0000	18	1808	0	1982	0.000185
	rs55813014	1267325	T	C	0.3313	0.3922	585	1225	664	1296	0.9999
	rs58125533	1267475	C	T	0.1886	0.4358	368	1428	340	1618	0.1415
	rs117757264	1267670	A	G	0.0158 *	0.2768	24	1802	36	1944	0.9981
	rs34528873	1269215	T	C	0.0082 *	1.0000	14	1798	17	1961	1
	rs4963055	1269398	C	T	0.4015	0.4656	679	1069	797	1131	0.9671
	rs2943517	1271321	C	G	0.3322	0.1536	575	1197	662	1290	0.9987
	rs2943512	1272226	A	C	0.3296	0.0989	561	1175	654	1296	1
	rs202131299	1272527	T	C	0.0187	1.0000	35	1785	36	1946	1
	rs3021155	1272709	A	G	0.0590	0.0296	96	1732	128	1840	0.9429
	rs3021156	1272754	G	A	0.0633	0.6192	103	1729	137	1821	0.8796
	rs55693520	1272800	T	C	0.0026 *	1.0000	5	1829	5	1975	1
	rs3829224	1276327	A	G	0.2732	0.1588	481	1351	561	1421	0.982
	rs199967813	1276738	A	G	0.0260	0.1632	3	1827	96	1882	1e-08
	rs55741856	1277953	A	G	0.0029 *	1.0000	8	1826	3	1977	0.9425
	rs192744525	1280193	A	G	0.0256	0.4094	14	1788	82	1864	1e-08
	rs55856616	1280238	A	G	0.0029 *	1.0000	6	1824	5	1975	1
	rs199785958	1282744	C	T	0.0250	0.2651	3	1827	92	1884	1e-08
*ABCC12*	rs200272726	48121966	T	C	0.0076*	1.0000	24	1708	4	1944	0.0008088
	rs7193955	48122582	G	A	0.1316	0.7967	216	1612	285	1695	0.3939
	rs34135219	48145742	A	T	0.0047*	1.0000	8	1818	10	1970	1

* Rare variants with MAF ≤ 0.0162.

** Empirical p-values from 100,000,000 permutation analyses.

**Fig 2:**
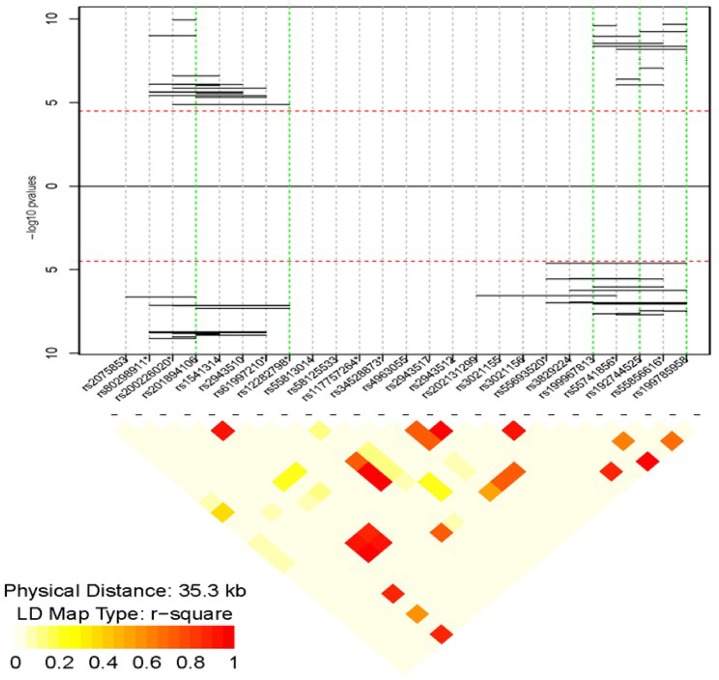
Haplotypes association results across *MUC5B*. the y axis represents–log_10_ (p values) and the x axis shows position within *MUC5B*. Red dotted lines indicate the Bonferroni correction level (-log_10_ (0.05/1,553)), Points above the line are odds ratio values > 1, and below are odds ratio values < 1. Green dotted lines indicate the positions of significantly associated SNPs in single SNPs analyses. The “*” symbol by the SNP label indicates rare variants (MAF ≤ 0.0162). The LD heat map presents pairwise r^2^ values within *MUC5B* based on the CADM study.

Based on the 25 markers available in the *MUC5B* gene (~35kb), we evaluated all possible 1,553 haplotypes for association with T2D. A total of 66 haplotypes showed significant association with T2D status (Bonferroni corrected p value of < 3.2×10^−5^; S3 Table and Fig 2). Each of the 66 haplotypes contained at least one SNP that showed single marker association with T2D (Table 3). For example, we observed 85 copies (4.39%) of the haplotype “CTGCCC” (Fig 2, amino acid positions from 1310 to 2836) among the controls compared to 3 copies (0.16%) among the T2D cases with a highly significant protective odd ratio (OR) of 0.031 (p-value 6.93×10^−8^). Also, there were 87 (4.49%) copies of the haplotype “AGAGC” (amino acid position from 5339 to 5732) among T2D cases compared to 3 (0.16%) copies among the controls (OR = 0.035, p-value 9.02×10^−8^). The partial correlation between these two haplotypes (CTGCCC and AGAGC) is 0.90. We observed that 2 (0.11%) copies of both CTGCCC and AGAGC haplotypes were present among those with T2D; while 79 (3.99%) copies of both CTGCCC and AGAGC were present among those without T2D, p value of 1.55×10^−6^ (OR and 95% C.I = 0.032 [0.008, 0.129]).

Three variants (2 rare: rs200272726, rs34135219; and one common: rs7193955) in *ABCC12* (ATP-binding cassette, sub-family C, member 12, GeneID: 94160, 16q12.1) had suggestive genome wide significant associations with T2D (Table 2). The MAF of rare variant rs200272726 (T allele), was 0.0141 for cases and 0.0020 for controls. The T allele of rs200272726 was significantly associated with T2D (empirical *p*-value = 8.01 × 10^−4^). The G allele of common variant rs7193955 was associated with T2D (empirical *p*-value = 0.04143) and had MAF was 0.1334 for cases and 0.1681 for controls (Table 3).

Genome-wide association studies (GWAS) for T2D [19–42] have identified 76 loci associated with T2D in East Asians. Based on the joint analysis of common and rare variants using SKAT, we evaluated the 46 gene sets available in our dataset. Six of the 46 gene sets (*CDKAL1*, *KCNJ11*, *KCNQ1*, *MPHOSPH9*, *PSMD6*, and *ZFAND6*) were replicated in the combined rare and common variants analysis (Table 4). In our analysis, there are 31,901 SNPs with MAF ≥ 0.016 (defined as common variants in SKAT). We replicated 2 (rs7754840, and rs4712524) of the 10 previously reported common *CDKAL1* SNPs for T2D in 15 East Asian GWAS or GWAS meta-analysis studies[19–21, 25, 29, 30, 32–39]. Also, we replicated 2 (rs2237897, and rs2237892) of the 7 previously reported SNPs in *KCNQ1* from 12 East Asian studies[19, 20, 24, 28–32, 36, 40–42]. Our local replication strategy (targeted SNP ± 250kb window) did not identify any significant association after adjustment for multiple comparisons.

**Table 4:** Replication of previous GWAS Findings in East Asian ancestry studies.

				Original Studies						CADM study	
										SKAT	Exact SNP *
PUBMED ID	Region	PPS	Genes	SNPs/Risk Allele	Context	Freq. (Risk Allele)	Reported P	OR/Beta	95%C.I.	p values	P value
24509480	6p23.3	20679478	CDKAL1	rs7756992/G	intron	0.260	2.00E-26	1.2	[1.16–1.25]	0.0347	
17460697	6p23.3	20679478	CDKAL1	rs7756992/G	intron	0.260	8.00E-09	1.2	[1.13–1.27]	0.0347	
23945395	6p23.3	20661019	CDKAL1	rs7754840/C	intron	0.420	2.00E-13	1.18	[1.13–1.23]	0.0347	0.0393
22961080	6p23.3	20661019	CDKAL1	rs7754840/C	intron	0.411	7.00E-10	1.35	[1.23–1.48]	0.0347	0.0393
17463246	6p23.3	20661019	CDKAL1	rs7754840/C	intron	0.310	4.00E-11	1.12	[1.08–1.16]	0.0347	0.0393
17463248	6p23.3	20661019	CDKAL1	rs7754840/C	intron	0.360	4.00E-11	1.12	[1.08–1.16]	0.0347	0.0393
22693455	6p23.3	20686342	CDKAL1	rs7766070/A	intron	0.270	7.00E-10	1.26	[1.17–1.35]	0.0347	
22693455	6p23.3	20686342	CDKAL1	rs7766070/A	intron	0.270	6.00E-11	1.21	[1.14–1.28]	0.0347	
21490949	6p23.3	20652486	CDKAL1	rs9295474/G	intron	0.360	9.00E-06	1.16	[1.09–1.24]	0.0347	
20581827	6p23.3	20687890	CDKAL1	rs10440833/A	intron		2.00E-22	1.25	[1.20–1.31]	0.0347	
19401414	6p23.3	20657333	CDKAL1	rs4712523/G	intron	0.410	7.00E-20	1.27	[1.21–1.33]	0.0347	
18711366	6p23.3	20657634	CDKAL1	rs4712524/G	intron	0.420	3.00E-10	1.22	[1.15–1.31]	0.0347	0.0459
18372903	6p23.3	20703721	CDKAL1	rs6931514/G	intron		1.00E-11	1.25	[1.17–1.33]	0.0347	
17463249	6p23.3	20660803	CDKAL1	rs10946398/C	intron	0.320	1.00E-08	1.16	[1.10–1.22]	0.0347	
17554300	6p23.3	20717024	CDKAL1	rs9465871/C	intron	0.180	3.00E-07	1.18	[1.04–1.34]	0.0347	
24509480	11p15.1	17387083	KCNJ11	rs5215/C	missense	0.380	3.00E-11	1.08	[1.04–1.12]	0.0474	
18372903	11p15.1	17387083	KCNJ11	rs5215/C	missense		4.00E-07	1.16	[1.09–1.23]	0.0474	
17463249	11p15.1	17387083	KCNJ11	rs5215/C	missense		5.00E-11	1.14	[1.10–1.19]	0.0474	
19056611	11p15.1	17388025	KCNJ11	rs5219/?	missense		5.00E-07	1.19	[1.11–1.27]	0.0474	
17463246	11p15.1	17388025	KCNJ11	rs5219/T	missense	0.470	1.00E-07	1.15	[1.09–1.21]	0.0474	
17463248	11p15.1	17388025	KCNJ11	rs5219/T	missense	0.460	7.00E-11	1.14	[1.10–1.19]	0.0474	
24509480	11p15.1	2825839	KCNQ1	rs163184/G	intron	0.500	2.00E-14	1.09	[1.04–1.13]	0.0459	
24390345	11p15.1	2837316	KCNQ1	rs2237897/C	intron		9.00E-15	1.31	[1.22–1.41]	0.0459	0.0047
18711366	11p15.1	2837316	KCNQ1	rs2237897/C	intron	0.340	1.00E-16	1.33	[1.24–1.41]	0.0459	0.0047
24101674	11p15.1	2810311	KCNQ1	rs8181588/A	intron	0.480	5.00E-09	1.3		0.0459	
23945395	11p15.1	2818521	KCNQ1	rs2237892/C	intron	0.610	4.00E-29	1.3	[1.24–1.36]	0.0459	0.0133
19401414	11p15.1	2818521	KCNQ1	rs2237892/C	intron	0.590	1.00E-26	1.33	[1.27–1.41]	0.0459	0.0133
22961080	11p15.1	2818521	KCNQ1	rs2237892/C	intron	0.657	1.00E-07	1.32	[1.19–1.46]	0.0459	0.0133
21573907	11p15.1	2818521	KCNQ1	rs2237892/C	intron		4.00E-06	1.2	[1.11–1.29]	0.0459	0.0133
18711367	11p15.1	2818521	KCNQ1	rs2237892/C	intron	0.610	2.00E-42	1.4	[1.34–1.47]	0.0459	0.0133
21799836	11p15.1	2822986	KCNQ1	rs163182/C	intron	0.340	2.00E-17	1.28		0.0459	
20581827	11p15.1	2670241	KCNQ1	rs231362/G	intron;ncRNA		3.00E-13	1.08	[1.06–1.10]	0.0459	
20174558	11p15.1	2835964	KCNQ1	rs2237895/C	intron	0.330	1.00E-09	1.29	[1.19–1.40]	0.0459	
24509480	12q24.31	123156306	MPHOSPH9	rs1727313/C	ncRNA		1.00E-08	1.06	[1.04–1.08]	0.0367	
22158537	3p14.1	64062621	PSMD6	rs831571/c		0.610	8.00E-11	1.09	[1.06–1.12]	0.0162	
20581827	15q25.1	80139880	ZFAND6	rs11634397/G			2.00E-09	1.06	[1.04–1.08]	0.0363	

*Empty cells: information not available

**Fig 3:**
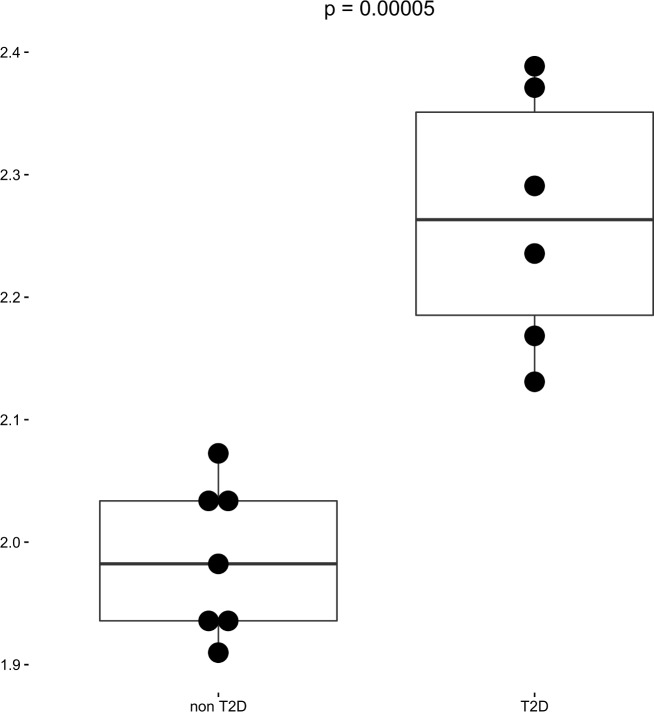
*MUC5B* differential expression in pancreatic islets from T2D and non-T2D organ donors. Displayed on the y axis are the mean and standard deviation values of log_2_ transformation of expression data.

Publicly available MIAME compliant gene expression data (GEO, accession number GSE25724; GDS3882; http://www.ncbi.nlm.nih.gov) generated from 13 pancreatic organ donors using the HG-U133A Affymetrix Chips was downloaded and evaluated for differential gene expression. Seven of the 13 donors did not have diabetes (mean age: 58 ± 17 years, gender: 4 males/3 females; mean BMI: 24.8 ± 2.5 kg/m^2^), and six had T2D (mean age: 71 ± 9 years; gender: 3 males/3 females; mean BMI: 26.0 ± 2.2 kg/m^2^). In a model that adjusted for sex, age and BMI, we observed significantly higher *MUC5B* expression in the group with T2D compared to those without diabetes (*p*-value = 0.00005; Fig 3).

## Discussion

We identified both rare and common variants within the *MUC5B* gene that were associated with T2D in this study conducted among Han Chinese. These results were replicated in a large sample of over 10,000 African ancestry individuals. We also identified several haplotypes within *MUC5B* that showed significant associations with T2D. Notably, individuals with T2D had significantly higher expression levels of *MUC5B* compared to those without T2D.

*MUC5B* encodes a member of the mucin family of proteins. These proteins are highly glycosylated macromolecular components[43]. As indicated above, the expression of *MUC5B* is increased among individuals with T2D compared to controls; however, the underlying mechanistic explanation driving the increased expression among diabetics has not been elucidated. Published studies suggest that the expression of MUC5B may be mediated through insulin-like growth factor-1 (IGF-1) and p38 mitogen-activated protein kinases (MAPK). *MUC5B* mRNA expression is induced by the action of IGF-1[44]. It has been reported that individuals with T2D, obesity, or both have increased levels of IGF-1[45–47] and that IGF-1 induced MUC5B expression is regulated by activation of p38 MAPK[44]. High levels of glucose have been shown to activate p38 MAPK signaling pathway in pancreatic β cells[48–50]. In animal studies, p38 has been shown to play an important role in diabetes-induced inflammation[51].

The lung is a target organ for T2D. Abnormal pulmonary function has been observed in individuals with T2D, the most consistent abnormalities include poor lung elasticity, reduced diffusion capacity due to impaired capillary blood volume, reduced absolute thoracic gas volumes, reduced lung volume and airflow resistance[52–54]. T2D may lead to abnormal pulmonary function through non-enzymatic glycosylation-induced alteration of the chest wall and bronchial tree collagen protein, which induces fibrous tissue formation, thickening of the basal lamina, increased protein catabolism, neuropathy of the phrenic nerve and diaphragmatic paralysis[54–57]. In healthy lungs, MUC5B is expressed in the goblet cells of bronchi and bronchioles. It has been found to be up-regulated in some human pulmonary diseases[58]. In a study of individuals with lung disease, a genome-wide linkage scan showed that a common promoter of *MUC5B* was associated with familial interstitial pneumonia and idiopathic pulmonary fibrosis; MUC5B was highly expressed among diseased individuals, compared to controls[59]. A recent meta-analysis that included Asian populations showed a strong association between *MUC5B* (rs35705950 polymorphism) and risk of idiopathic pulmonary fibrosis[60]. The diabetes status of the individuals in the study was not stated.

The *MUC5B* gene is composed of tandem repeats which are flanked by cysteine-rich subdomains (845 residues upstream and 700 residues downstream). The cysteine-rich subdomains were similar to the D-domains of human pro-Von Willebrand factor[61, 62]. Increased levels of von Willebrand factor, an indication of damage to endothelial cells, have been showed association with diabetes[63]. It also reported as a predictive markers for diabetic nephropathy and neuropathy, thus providing a clue that endothelial dysfunction precedes the onset of diabetic microangiopathy[63]. In previous studies of Sjögren's syndrome, a chronic autoimmune disease in which the body’s white blood cells destroy the exocrine glands, a relationship between *MUC5B*, von Willebrand factor and diabetes was suggested[64, 65], indicating a potential role of *MUC5B* in cardiovascular complications of T2D. An NF-kappa-B binding site in the *MUC5B* promoter showed that activation of the NF-kappa-B signaling pathway upregulated *MUC5B* mRNA expression 2 fold[66]. NF-kappa-B signaling pathway plays an important role in immune and inflammatory response[67], supporting a potential role of *MUC5B* in T2D.

## Conclusions

We identified rare and common variants in the *MUC5B* gene that are associated with T2D in Han Chinese. Our findings suggest that dysregulated *MUC5B* expression may be involved in the pathogenesis of T2D. As a strong candidate gene for T2D, *MUC5B* may play an important role in the mechanisms underlying T2D etiology and its complications.

## Supporting information

S1 Fig QQ plots exome array association results. The y axis represents observed -log_e_ (p values), and the x axis is expected -log_e_ (p values).
(TIFF)

S1 Table Basic characteristics of the study participants by type 2 diabetes status in African ancestry replication study. (XLSX)

S2 Table Description of SNPs in the discovery and replication analyses. (XLSX)

S3 Table *MUC5B* haplotype frequencies and associations with T2D. (XLSX)
