# Supplementary figures and images for: Common and rare exonic MUC5B variants associated with type 2 diabetes in Han Chinese
Source: PLoS One. 2017 Mar 27;12(3):e0173784. doi: 10.1371/journal.pone.0173784 (PMC5367689; doi:10.1371/journal.pone.0173784)

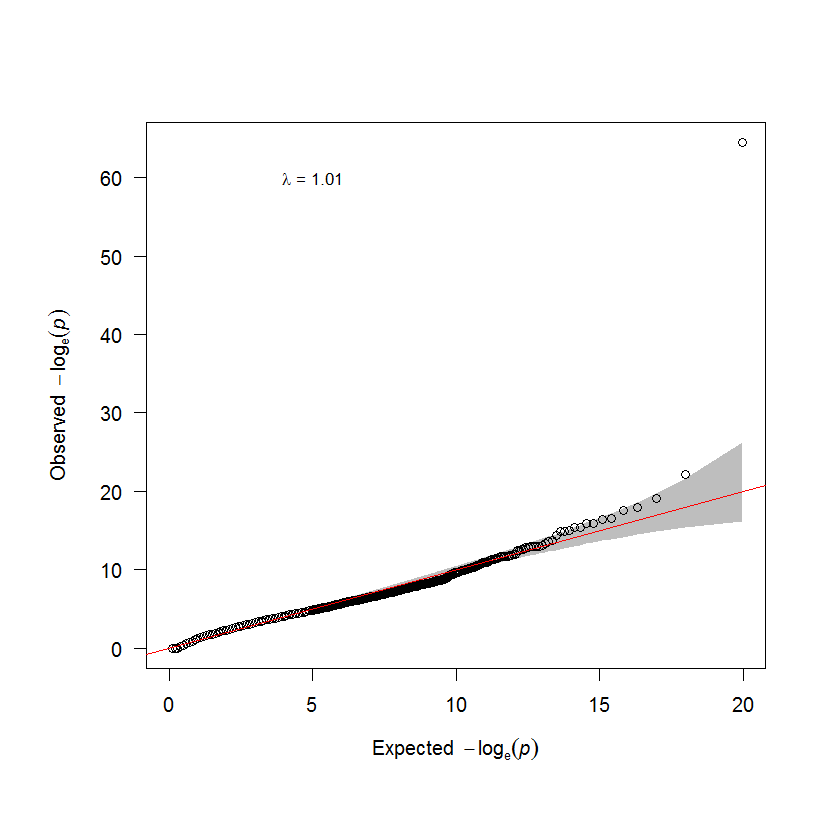

Supplement: S1 Fig — The y axis represents observed -loge (p values), and the x axis is expected -loge (p values). (TIFF) [file pone.0173784.s001.tiff]
